# The metabolic spatial covariance pattern of definite idiopathic normal pressure hydrocephalus: an FDG PET study with principal components analysis

**DOI:** 10.1186/s13195-023-01339-x

**Published:** 2023-11-18

**Authors:** Alexander Rau, Nils Schröter, Ganna Blazhenets, Christoph Maurer, Horst Urbach, Philipp T. Meyer, Lars Frings

**Affiliations:** 1 Department of Neuroradiology, Medical Center - University of Freiburg and Faculty of Medicine, University of Freiburg, Freiburg, Germany; 2 Department of Diagnostic and Interventional Radiology, Medical Center - University of Freiburg and Faculty of Medicine, University of Freiburg, Freiburg, Germany; 3 Department of Neurology, Medical Center - University of Freiburg and Faculty of Medicine, University of Freiburg, Freiburg, Germany; 4 Department of Nuclear Medicine, Medical Center - University of Freiburg and Faculty of Medicine, University of Freiburg, Freiburg, Germany; 5 Center for Geriatrics and Gerontology, Medical Center - University of Freiburg and Faculty of Medicine, University of Freiburg, Freiburg, Germany

**Keywords:** Hydrocephalus, Normal pressure, Positron emission tomography-computed tomography, Dementia, Movement disorders

## Abstract

Identification of patients with idiopathic normal pressure hydrocephalus (iNPH) in a collective with suspected neurodegenerative disease is essential. This study aimed to determine the metabolic spatial covariance pattern of iNPH on FDG PET using an established technique based on scaled subprofile model principal components analysis (SSM-PCA).

We identified 11 patients with definite iNPH. By applying SSM-PCA to the FDG PET data, they were compared to 48 age-matched healthy controls to determine the whole-brain voxel-wise metabolic spatial covariance pattern of definite iNPH (iNPH-related pattern, iNPHRP). The iNPHRP score was compared between groups of patients with definite iNPH, possible iNPH (*N* = 34), Alzheimer’s (AD, *N* = 38), and Parkinson’s disease (PD, *N* = 35) applying pairwise Mann–Whitney *U* tests and correction for multiple comparisons.

SSM-PCA of FDG PET revealed an iNPHRP that is characterized by relative negative voxel weights at the vicinity of the lateral ventricles and relative positive weights in the paracentral midline region. The iNPHRP scores of patients with definite iNPH were substantially higher than in patients with AD and PD (both *p* < 0.05) and non-significantly higher than those of patients with possible iNPH. Subject scores of the iNPHRP discriminated definite iNPH from AD and PD with 96% and 100% accuracy and possible iNPH from AD and PD with 83% and 86% accuracy.

We defined a novel metabolic spatial covariance pattern of iNPH that might facilitate the differential diagnosis of iNPH versus other neurodegenerative disorders. The knowledge of iNPH-associated alterations in the cerebral glucose metabolism is of high relevance as iNPH constitutes an important differential diagnosis to dementia and movement disorders.

**Supplementary Information:**

The online version contains supplementary material available at 10.1186/s13195-023-01339-x.

## Key points

• Subprofile model principal components analysis revealed a distinct metabolic spatial covariance pattern of definite idiopathic normal pressure hydrocephalus (iNPH).

• This iNPH-related pattern delineates patients with definite iNPH from patients with Alzheimer’s or Parkinson’s disease.

• The iNPH-related pattern expression was higher in definite than in probable iNPH.

**Supplementary Information:**

The online version contains supplementary material available at 10.1186/s13195-023-01339-x.

## Introduction

Idiopathic normal pressure hydrocephalus (iNPH) is clinically characterized by movement and cognitive disturbance as well as urinary incontinence, forming the typical “Hakim triad” [1]. Other neurodegenerative conditions such as Alzheimer’s disease (AD) or Parkinson’s disease (PD) can present similar clinical symptoms and thus hamper differential diagnoses. Since symptoms in iNPH are potentially reversible, e.g., by ventriculoperitoneal cerebrospinal fluid (CSF) shunting, identification of patients with iNPH is essential [2].

Magnetic resonance imaging (MRI)-based signs of iNPH are incorporated in current diagnostic criteria of iNPH [3]. The established pattern on MRI includes enlargement of the ventricles and the Sylvian fissure and constricted cerebrospinal fluid spaces in the high convexities of the brain (“tight high convexity”) leading to the so-called disproportionately enlarged subarachnoid space hydrocephalus (DESH) and a decreased callosal angle [4–7].

In the workup of suspected iNPH expert opinion recommends FDG PET for the detection of typical patterns of potentially co-existing neurodegenerative diseases which might cause part of the symptoms [8]. Moreover, there is evidence of altered cerebral metabolic activity in patients with iNPH: Previous molecular imaging studies with positron emission tomography (PET) and [^18^F]fluorodeoxyglucose (FDG) showed *decreases in* glucose metabolism in iNPH either wide-spread [9] or accentuated in the vicinity of the lateral ventricles [10]. Additionally, Ohmichi and colleagues [11] used perfusion single-photon emission computed tomography (SPECT) to describe the so-called convexity apparent hyperperfusion (CAPPAH) sign, i.e., *increased* cerebral blood flow at the high convexity.

Considering previous observations [10, 11], we applied scaled subprofile model principal components analysis (SSM-PCA) to examine whether there is an iNPH-related spatial covariance pattern (iNPHRP) that would comprise regions with both relative increased and decreased weight of cerebral glucose metabolism. SSM-PCA is a powerful voxel-wise analysis method to reduce the dimensionality of FDG PET data that has previously been applied to study various diseases like parkinsonian syndromes [12–14], dementia [15, 16] as well as non-neurodegenerative disorders [17]. It allows to determine the degree of similarity between a metabolic spatial covariance pattern and each individual’s FDG PET data resulting in a one-score-per-subject rating. We hypothesize that such a pattern and patients’ scores would not only distinguish iNPH from healthy controls, but also from other neurodegenerative conditions like AD or PD, which is of high clinical relevance.

## Material and methods

### Patient cohort

The present retrospective study was approved by the local ethics committee (Ethics committee, University of Freiburg, application No. 22/20) and carried out in accordance with the declaration of Helsinki and its later amendments. Written informed consent was waived.

From the clinical registry of patients assessed for suspected neurodegenerative diseases between 2010 and 2020 at our tertiary referral center, we identified all patients over 60 years of age who had received both cerebral FDG PET and MRI within a 6-month period. Only patients with DESH on visual reading of the MRI scans were included. Imaging data with motion artifacts were omitted from further analysis.

Of these patients with DESH, we identified those who presented with more than one clinical symptom of the Hakim triad that was not explained by other diseases. Within this group, clinical records were screened to detect shunt responders, who constitute to the primary target group corresponding to definite iNPH according to the current diagnostic guidelines [3]. Patients without CSF shunting were assigned to another group corresponding to possible iNPH. In patients with CSF shunting, all analyzed MRI and FDG PET data were acquired before the shunt procedure.

FDG PET from healthy elderly controls (HEC, *N* = 48), acquired on the same PET/CT systems as the patient data (see below), served as control group data. As additional patient groups, we further enrolled subjects with clinical diagnosis of AD (*N* = 38) and PD (*N* = 35) who had received FDG PET for assessment of a suspected neurodegenerative disease. Diagnoses of AD and PD were made retrospectively according to the respective consensus criteria [18, 19] based on all available data including CSF and imaging findings (i.e., CT, MRI, PET with FDG, amyloid and tau ligands, and dopamine transporter SPECT, as available) as best clinical lifetime diagnosis. Neither of these groups (HEC, AD, PD) included subjects with signs of iNPH on MRI.

### MRI data acquisition and analysis

MRI data was acquired with 3D T1-weighted datasets with an isotropic voxel size of 1 mm at both 1.5 and 3.0 Tesla. Visual reading of the MRI scans was performed by a neuroradiologist (A.R., four years of experience in neuroimaging). Patients with clear evidence of pathologies known to cause secondary NPH, such as stroke or meningitis, were excluded. Human reading was supported using a recently developed and validated support vector machine algorithm (SVM) [20].

### Clinical data

Clinical files of all patients with DESH were screened for iNPH symptoms, especially gait disturbance, incontinence and cognitive impairment, and effects of CSF shunting — if performed — by an experienced neurologist (N.S., 6 years of experience in neurodegenerative disorders).

### FDG PET acquisition and processing

FDG PET scans were acquired on “TF Gemini” or “Vereos” PET/CT systems (Philips Healthcare, Amsterdam, Netherlands). A static 10-min PET scan was acquired 50 min after injection of 214 ± 22 MBq (Gemini) or 211 ± 19 MBq of FDG (Vereos) under resting conditions at ambient noise and with eyes open. Fully corrected PET datasets were reconstructed as previously described [21, 22]. Acquisition protocols were identical for all FDG PET acquired on the respective system.

FDG PET data were preprocessed using Matlab R2018b (Mathworks, Natick, MA) and statistical parametric mapping 12 (SPM12; www.fil.ucl.ac.uk/spm): reconstructed PET scans were normalized to an in-house template in Montreal Neurological Institute (MNI) space (2 mm isotropic voxel size; SPM12 default normalization settings; “old normalise”). FDG PET data were smoothed with a Gaussian kernel of 9 mm (Vereos) or 8 mm (Gemini) full width at half maximum (FWHM) in order to achieve a comparable spatial resolution.

### Statistical analysis

A voxel-based PCA was carried out to construct the metabolic spatial covariance pattern related to definite iNPH. This approach has the advantage of combining multiple, interacting brain regions of metabolic increases and decreases into a whole-brain pattern in a data-driven fashion. To generate such a spatial covariance pattern, the scaled subprofile model PCA (SSM-PCA, [23]) was applied using the ScAnVP/SSM-PCA toolbox to discriminate between definite iNPH and HEC as previously described [15, 24]. Each individual’s expression score of the iNPH-related spatial covariance pattern was evaluated by the topographic profile rating algorithm involving computation of the internal vector product of the subject’s residual profile vector and the pattern vector [25] as implemented in the ScAnVP/SSM-PCA toolbox.

Individual iNPHRP scores were subsequently derived for all patients and controls and z-transformed with respect to controls’ iNPHRP scores. Scores of the definite iNPH group were compared in a pairwise fashion to those of the possible iNPH, AD, and PD groups applying Mann–Whitney *U* tests. Likewise, the iNPHRP scores of the possible iNPH group were compared to those of the AD and PD groups.

We employed a cut-off derived from a receiver operating characteristics (ROC) analysis (definite iNPH versus HEC; criterion: best Youden Index) to calculate accuracy, sensitivity, and specificity of iNPHRP scores for the discrimination of (a) definite iNPH from possible iNPH, AD, and PD groups and (b) possible iNPH from AD and PD.

In order to test the influence of possible confounders, we computed an ANCOVA with factor “group” and covariates “sex,” “age,” and “PET/CT system” and post hoc Tukey tests.

Statistical analyses were performed using R 4.0.3 (https://www.R-project.org/).

### Data availability statement

The data that support the findings of this study are available on request from the corresponding author. The data are not publicly available as they contain information that could compromise the privacy of research participants.

## Results

We identified 11 patients with definite iNPH (CSF shunt responders) and 34 patients with possible iNPH (DESH and more than one clinical symptom of the Hakim triad, see Table 1).

**Table 1:** Participant characteristics

	Definite iNPH	Possible iNPH	AD	PD	HEC
*N*	11	34	38	35	48
iNPRP score (median, IQR)	3.16 [2.48, 4.02]	2.31 [1.76, 2.98]	− 0.53 [− 0.93, 0,23]	− 0.77 [− 1.01, − 0.58]	− 0.06 [− 0.74, 0.46]
Age (years, mean, and S.D.)	71.9 (7.6)	73.6 (7.0)	67.5 (9.1)	65.3 (9.0)	75.1 (7.4)
Sex (m:f)	8:3	24:10	21:17	23:12	21:27
PET/CT system (Gemini:Vereos)	7:4	21:13	4:34	4:31	35:13
Gait disturbance (yes:no)	10:1	34:0	NA	NA	NA
Urinary incontinence (yes:no)	8:3	16:11 (NA in 7 cases)	NA	NA	NA
Cognitive impairment (yes:no)	11:0	31:2 (NA in 1 case)	NA	NA	NA

*Abbreviations*: *iNPH* idiopathic normal pressure hydrocephalus, *iNPHRP* idiopathic normal pressure hydrocephalus-related pattern, *AD* Alzheimer’s disease, *PD* Parkinson’s disease, *HEC* healthy elderly controls, *NA* not available

**Fig. 1:**
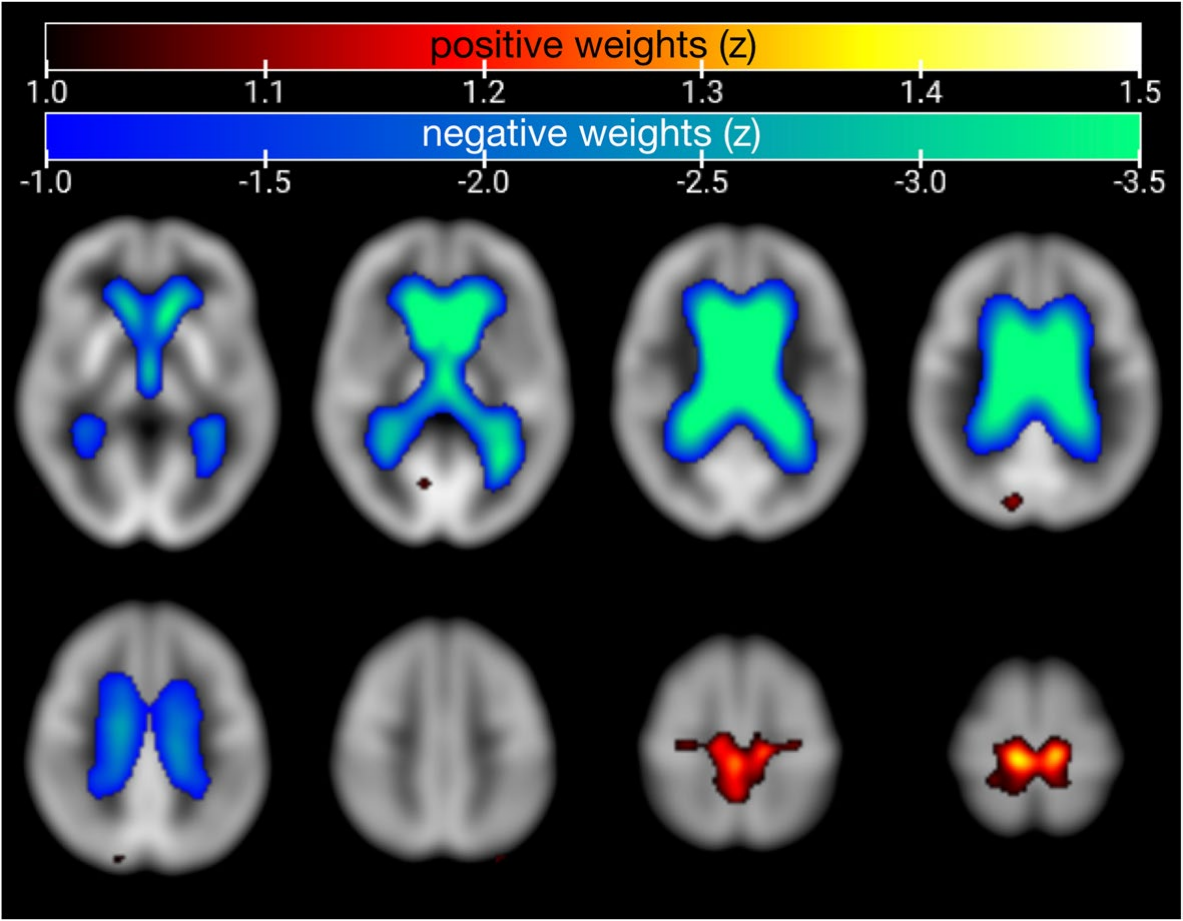
SSM-PCA-based metabolic covariance pattern of iNPH as assessed in patients with definite iNPH (*N* = 11) versus healthy elderly controls (*N* = 48), superimposed on axial slices of an in-house FDG PET template in MNI space. Threshold for visualization purposes at *z* > 1.0 for positive weights and *z* <  − 1.0 for negative weights. Image depicted in radiological orientation, i.e., the left image side corresponds to the patient’s right body side

The SSM-PCA group analysis identified 59 principal components among which principal component 1 accounted for 33.69% of the variance in the data and was the only component that showed a group difference between patients with definite iNPH and HEC (*p* < 0.001, AUC ROC = 0.99). The iNPHRP includes voxels with positive voxel weights (corresponding to the relative increase of glucose metabolism, see Online Resource 2) mainly at the paracentral lobule and negative voxel weights (relative metabolic decreases, see Online Resource 2) at the vicinity of the lateral ventricles including the caudate nucleus, thalamus, and the cingulum (Fig. 1).

**Fig. 2:**
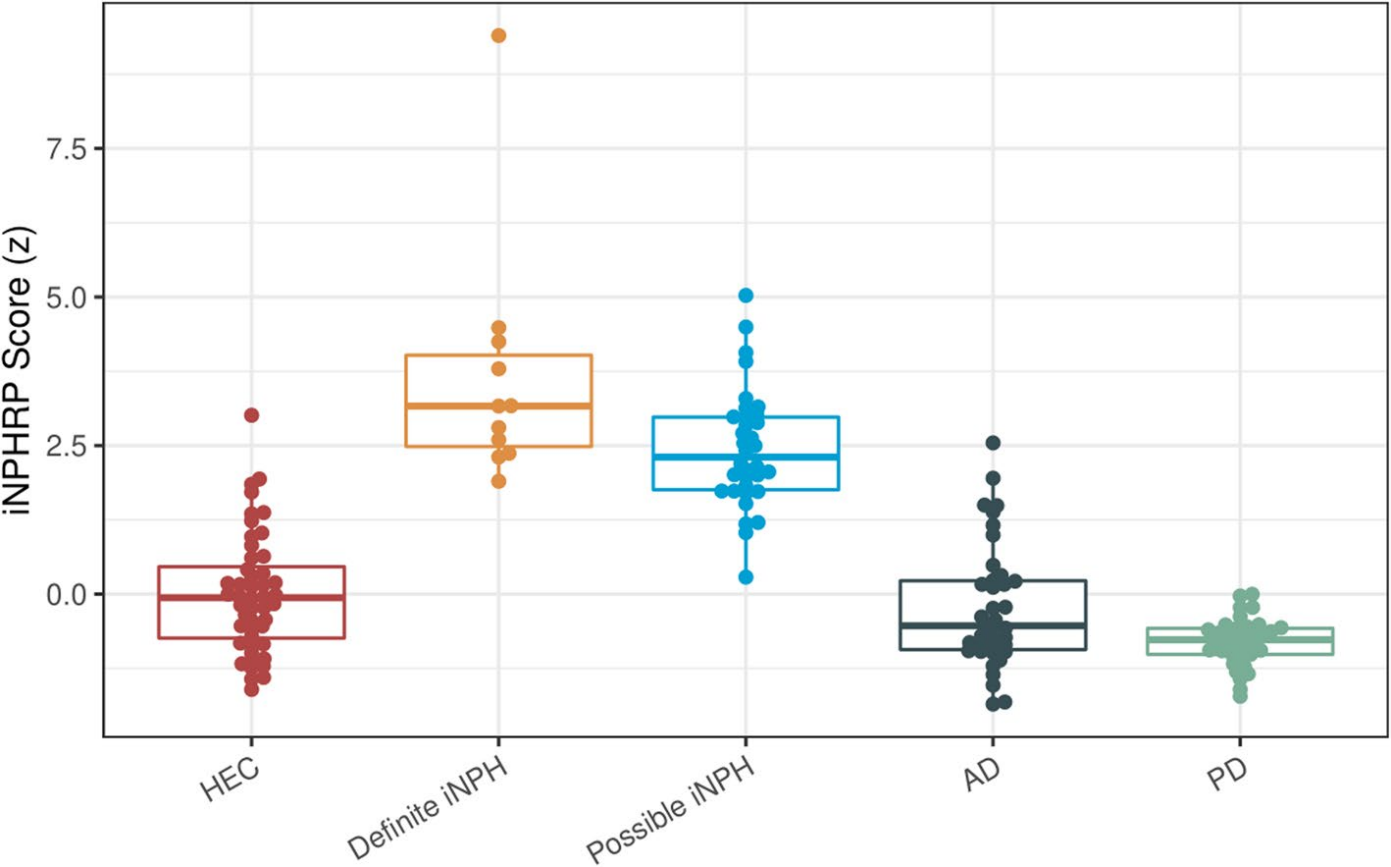
Boxplots of iNPHRP scores by group. HEC, healthy elderly controls; iNPH, idiopathic normal pressure hydrocephalus; AD, Alzheimer’s disease; PD, Parkinson’s disease

Pairwise Mann–Whitney *U* tests showed that the resulting iNPHRP scores in definite iNPH were significantly higher than in AD and PD (*p* < 0.05, Bonferroni-corrected for multiple comparisons). Scores in definite iNPH were also numerically higher than in possible iNPH, although they failed to reach significance after Bonferroni correction. In possible iNPH, iNPHRP scores were significantly higher than in AD and PD (Bonferroni-corrected *p* < 0.05; see Fig. 2, Table 1, and Online Resource 1).

When the ROC-derived cut-off (iNPHRP score ≥ 26.57) was used as a threshold, the iNPHRP score discriminated definite iNPH from AD and PD with an accuracy of 96% and 100%, respectively. Using the same threshold, iNPHRP scores also discriminated possible iNPH from AD (accuracy of 83%) and PD (accuracy of 86%; please see Online Resource 1).

The confounder analysis with covariates “age,” “sex,” and “PET/CT system” essentially replicated these results: the main effect of factor “group” was highly significant (*F*(4,158) = 87.0, *p* < 0.001), with significantly higher iNPHRP scores in definite iNPH than in possible iNPH (adjusted *p* < 0.005), AD, PD, and HEC (adjusted *p* < 0.001) and significantly higher iNPHRP scores in possible iNPH than in AD, PD, and HEC (all adjusted *p* < 0.001). Partial eta^2^ revealed that the group factor explained more variance in the data (partial eta^2^ = 0.69) than the other variables (age, partial eta^2^ = 0.08; sex, partial eta^2^ = 0.02; PET/CT system, partial eta^2^ = 0.03).

Exploratory post hoc analyses of the relationship between iNPHRP scores and clinical features of iNPH showed that iNPH patients with cognitive impairment had higher iNPHRP scores (*z* = 2.77 + / − 1.46) than those without (2.03 + / − 0.04; *p* = 0.002, *t* test). In patients with definite or possible iNPH, higher iNPHRP scores were associated (at trend level) with more time needed to accomplish the timed-up-and-go test (*r* = 0.45, *p* = 0.082, available in 16/45 iNPH patients), but not 15 m walking (*N* = 18/45) or the five-chair test (*N* = 13/45).

## Discussion

We determined the metabolic spatial covariance pattern of definite iNPH on FDG PET in comparison with healthy elderly controls. Subject scores of iNPHRP were significantly higher in definite iNPH than in AD and PD patients, allowing for a discrimination between the groups with an accuracy of 96% and 100%, respectively, which is of high differential diagnostic importance.

The constructed metabolic covariance pattern of definite iNPH comprises regions with relative increases as well as decreases of metabolism (see Online Resource 2). We assume that the relative *hypo*metabolism around the lateral ventricles mainly reflects the typical ventriculomegaly in iNPH and therefore not an altered glucose metabolism per se, but rather an altered ratio of brain parenchyma to CSF. Hence, less regional FDG uptake at the caudate nucleus might rather be due to this partial volume effect. Therefore, we assume that other imaging modalities, like DAT SPECT, might also detect abnormalities in this region [26]. We interpret the relative *hyper*metabolism in the paracentral midline region in iNPH to be caused by a mixture of partial volume effects, i.e., the narrowing of the sulci and subarachnoid space over the high-convexity/midline surface on one hand, and on the other hand relatively preserved neuronal function in this area in the presence of impaired neuronal function in other areas of the brain. Hence, the relative *hyper*metabolism in this region in our view does not indicate increased glucose metabolism of brain parenchyma. Taken together, the metabolic covariance pattern of definite iNPH parallels the morphologic changes in definite iNPH with an emphasis on the ventricular enlargement and high-convexity/midline tightness (as corroborated by MRI data in Online Resource 3). This pattern might, beyond the morphologic changes, also reflect true metabolic changes, e.g. in the caudate nucleus. Caudate hypometabolism has previously been reported in iNPH [10], and the caudate nucleus has been linked to typical iNPH symptoms such as gait disturbance, cognitive impairment, and apathy [3].

Decreased cerebral glucose metabolism in iNPH has been described before: globally [27, 28] and in the vicinity of the lateral ventricles, comprising the striatum [10, 29]. By contrast, increased metabolism in iNPH was only reported in a volume-of-interest (VOI) based analysis [29]: The authors observed higher FDG uptake compared to healthy controls in a medial parietal VOI, but only in asymptomatic individuals with signs of iNPH on CT, but not in the group of patients with imaging and clinical features of iNPH. We postulate that increased FDG uptake could be observed in the medial parietal VOI due to its close proximity to the paracentral lobule, which was identified as the region of maximum positive weight (relatively increased metabolism) on the iNPHRP in our study. As VOI-based approaches rely on a priori assumptions, information might be lost by omitting certain regions in contrast to voxel-based approaches. Results of our whole-brain voxel-wise analysis strongly suggest to regard relative hypermetabolism of the paracentral region as an FDG PET indicator of iNPH.

Relative metabolic increase in the paracentral region closely resembles the CAPPAH sign on perfusion SPECT [11], though the current study introduces substantial improvement and novelty: (A) It provides a whole-brain, voxel-wise pattern, which can be expressed in a single numeric value per patient. (B) The iNPHRP also comprises relative decreases at the lateral ventricles, whereas the CAPPAH sign consists only of the hyperperfusion at the high convexity. (C) The iNPHRP was statistically derived in an observer-independent, data-driven fashion from definite iNPH patients. By contrast, the CAPPAH sign was presented after its observation in clinical routine, and the higher prevalence of iNPH in individuals with the CAPPAH sign was only secondarily confirmed. (D) The iNPHRP achieved a slightly better discrimination of iNPH patients from healthy elderly controls (sensitivity of 100% (11/11) and specificity of 96% (46/48)) compared to the CAPPAH’s sensitivity of 80% (24/30) and specificity of 100% (19/19)). (E) The SSM-PCA-based approach permits the calculation of individual subject scores of the iNPHRP as continuous values whereas the visual read of the CAPPAH sign only allows dichotomous classification. Thus, iNPHRP scores may be particularly suitable for prediction models and follow-up examinations. The iNPHRP score might hence convey information about disease severity, which needs to be addressed by further studies. (F) Finally, implementation of the SSM-PCA methodology and read-out of disease-related metabolic covariance pattern can easily be implemented in clinical routine and does not depend on rater experience, and therefore may assist less experienced raters. Of note, for clinical purposes, it may be advisable to read out and combine the spatial covariance patterns of several diseases of interest (e.g., iNPH-, PD-, and AD-related patterns), which has been shown to be a very powerful diagnostic approach in other clinical scenarios such as differential diagnosis of parkinsonian syndromes [14].

Individual iNPHRP scores could be calculated not only in patients from the derivation sample (definite iNPH), but, importantly, also in patients from other samples with overlapping symptoms (e.g. AD, PD). With diagnostic accuracies for iNPH of 100% against PD and 96% against AD, we strongly advocate for the use of FDG PET in the workup of neurodegenerative conditions to assess the presence of the metabolic iNPH spatial covariance pattern. This underlines its potential for clinical application in individual cases.

All iNPH patients analyzed in this study had DESH. Therefore, the identified iNPHRP might apply to iNPH with DESH specifically. According to current guidelines, a substantial proportion of definite iNPH might not have DESH [3]. Whether the iNPHRP is sufficiently sensitive to detect non-DESH iNPH needs to be addressed by further studies. Still, it might be considered useful in clinical routine to have the iNPHRP as an additional DESH marker, as DESH is among the best presurgical neuroimaging predictors of shunt response and to date only assessed dichotomously and subjectively [3, 30]. The possible iNPH group showed overlapping, but still numerically lower average iNPHRP scores than definite iNPH. Due to the retrospective character of this study, the further course of the patients in the possible iNPH group could not be systematically assessed. Therefore, a potential association between iNPHRP scores and shunt responsiveness in these could not be evaluated. This needs to be addressed in further studies, together with the reversibility of the observed spatial covariance pattern and its correlation with clinical symptoms’ expression. Other limitations of this study include the small sample size of definite iNPH cases and a lack of control groups with mixed diseases (e.g., AD plus iNPH). Moreover, the AD and PD groups were younger than the iNPH group, though age did not contribute substantially to the group differences in iNPHRP scores. A final limitation of this retrospective study is the sparse clinical data characterizing the iNPH patients: While documented data were missing in many cases, we did observe a relationship between higher iNPHRP scores and cognitive as well as motor impairment.

## Conclusion

We defined a novel metabolic spatial covariance pattern of iNPH that might facilitate the differential diagnosis of iNPH versus other neurodegenerative disorders. Further validation in prospective cohorts is needed, including correlation studies with clinical features, possible prediction of shunt response, and pattern reversibility.

### Supplementary Information

**Additional file 1: Online Resource 1.** Results from pairwise comparisons of iNPHRP scores between groups. **Online Resource 2.** In order to relate positive and negative voxel weights of the iNPHRP to alterations of normalized regional glucose metabolism in definite iNPH, FDG uptake (reference: brain parenchyma) was read out from all FDG PET at the positive and negative peak voxels of the iNPHRP (sphere of 4 mm radius), respectively. Plots confirmed that FDG uptake at the lateral ventricle (peak of negative voxel weights) was lowest among all groups in definite iNPH, whereas FDG uptake was highest in definite iNPH at the peak of positive voxel weights. **Online Resource 3.** Comparison between the relative increases of CSF and gray matter in definite iNPH (obtained from MRI) and the FDG PET-derived iNPHRP in the same subjects. Top, t values from group comparisons of CSF and gray matter volume between definite iNPH patients and HEC (separate SPM12 t tests). Bottom, iNPHRP positive and negative voxel weights.

## Data Availability

The data that support the findings of this study are available on request from the corresponding author. The data are not publicly available as they contain information that could compromise the privacy of research participants.

## Abbreviations

AD: Alzheimer’s disease
AUC ROC: Area under the receiver operating characteristic curve
CAPPAH: Convexity apparent hyperperfusion
CSF: Cerebrospinal fluid
DESH: Disproportionately enlarged subarachnoid space hydrocephalus
HEC: Healthy elderly controls
iNPH: Idiopathic normal pressure hydrocephalus
iNPHRP: Idiopathic normal pressure hydrocephalus-related pattern
PCA: Principal components analysis
PD: Parkinson’s disease
