# Supplementary material for: The metabolic spatial covariance pattern of definite idiopathic normal pressure hydrocephalus: an FDG PET study with principal components analysis
Source: Alzheimers Res Ther. 2023 Nov 18;15:202. doi: 10.1186/s13195-023-01339-x (PMC10657637; doi:10.1186/s13195-023-01339-x)
Supplement: Supplementary file 1 — Additional file 1: Online Resource 1. Results from pairwise comparisons of iNPHRP scores between groups. Online Resource 2. In order to relate positive and negative voxel weights of the iNPHRP to alterations of normalized regional glucose metabolism in definite iNPH, FDG uptake (reference: brain parenchyma) was read out from all FDG PET at the positive and negative peak voxels of the iNPHRP (sphere of 4 mm radius), respectively. Plots confirmed that FDG uptake at the lateral ventricle (peak of negative voxel weights) was lowest among all groups in definite iNPH, whereas FDG uptake was highest in definite iNPH at the peak of positive voxel weights. Online Resource 3. Comparison between the relative increases of CSF and gray matter in definite iNPH (obtained from MRI) and the FDG PET-derived iNPHRP in the same subjects. Top, t values from group comparisons of CSF and gray matter volume between definite iNPH patients and HEC (separate SPM12 t tests). Bottom, iNPHRP positive and negative voxel weights. [file 13195_2023_1339_MOESM1_ESM.docx]

**The metabolic spatial covariance pattern of definite idiopathic normal pressure hydrocephalus: An FDG PET study with principal components analysis**

**Online Resource 1** Results from pairwise comparisons of iNPHRP scores between groups.

|  | Definite iNPH | Possible iNPH | AD | PD | HEC |
| --- | --- | --- | --- | --- | --- |
| Definite iNPH | - |  |  |  |  |
| Possible iNPH | p = 0.024  d = 0.92  Acc = 0.79  Se = 0.91  Sp = 0.5 | - |  |  |  |
| AD | p < 0.001*  d = 2.91  Acc = 0.96  Se = 1  Sp = 0.95 | p < 0.001*  d = 2.60  Acc = 0.83  Se = 0.71  Sp = 0.95 | - |  |  |
| PD | p < 0.001*  d = 4.25  Acc = 1  Se = 1  Sp = 1 | p < 0.001*  d = 4.29  Acc = 0.86  Se = 0.71  Sp = 1 | p = 0.022  d = 0.72 | - |  |
| HEC | p < 0.001*  d = 2.91  Acc = 0.97  Se = 1  Sp = 0.96 | p < 0.001*  d = 2.44  Acc = 0.85  Se = 0.71  Sp = 0.96 | p = 0.265  d = 0.22 | p < 0.001*  d = 1.0 | - |

p, p value from Mann-Whitney U test (not corrected for multiple comparisons); * significant at Bonferroni-corrected p < 0.05; d, Cohen’s d; Acc, Se, and Sp describe the diagnostic accuracy, sensitivity, and specificity of iNPHRP scores at a given cut-off (iNPHRP score = 26.57, determined by ROC analysis with definite iNPH vs HEC; criterion: best Youden index) to discriminate definite iNPH and possible iNPH from other groups

**Online Resource 2**

In order to relate positive and negative voxel weights of the iNPHRP to alterations of normalized regional glucose metabolism in definite iNPH, FDG uptake (reference: brain parenchyma) was read out from all FDG PET at the positive and negative peak voxels of the iNPHRP (sphere of 4 mm radius), respectively. Plots confirmed that FDG uptake at the lateral ventricle (peak of negative voxel weights) was lowest among all groups in definite iNPH, whereas FDG uptake was highest in definite iNPH at the peak of positive voxel weights.


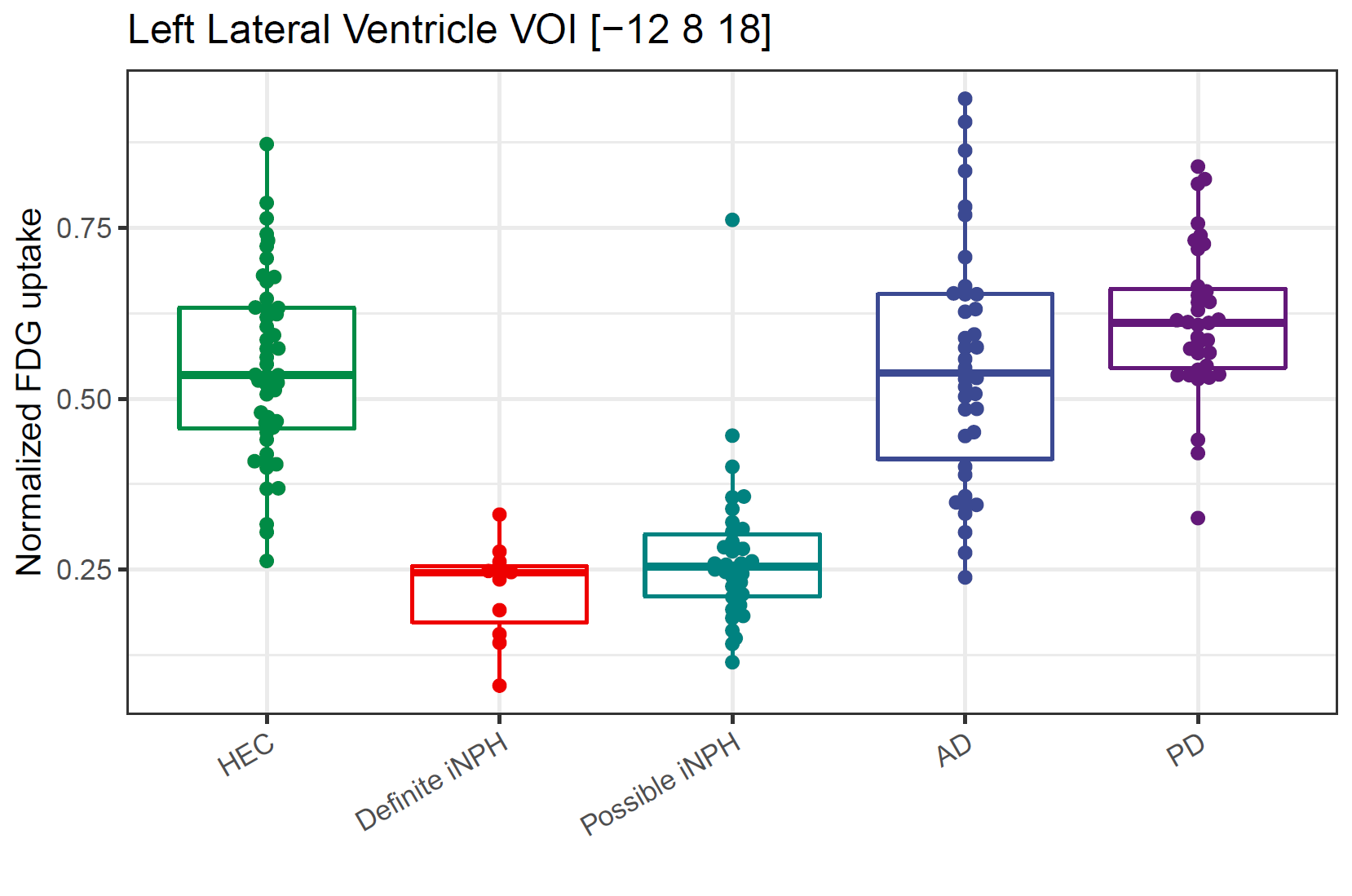


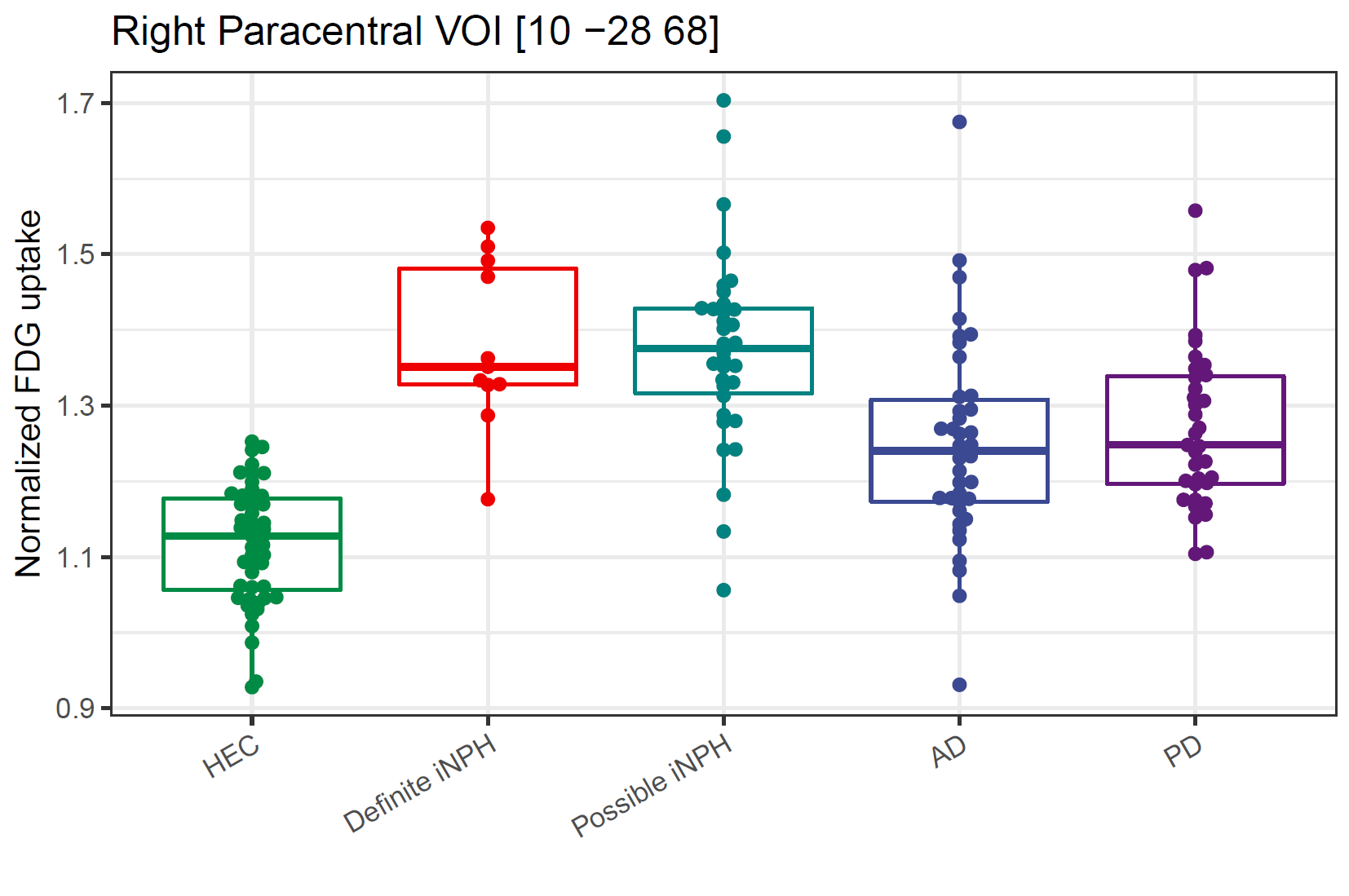


**Online Resource 3**


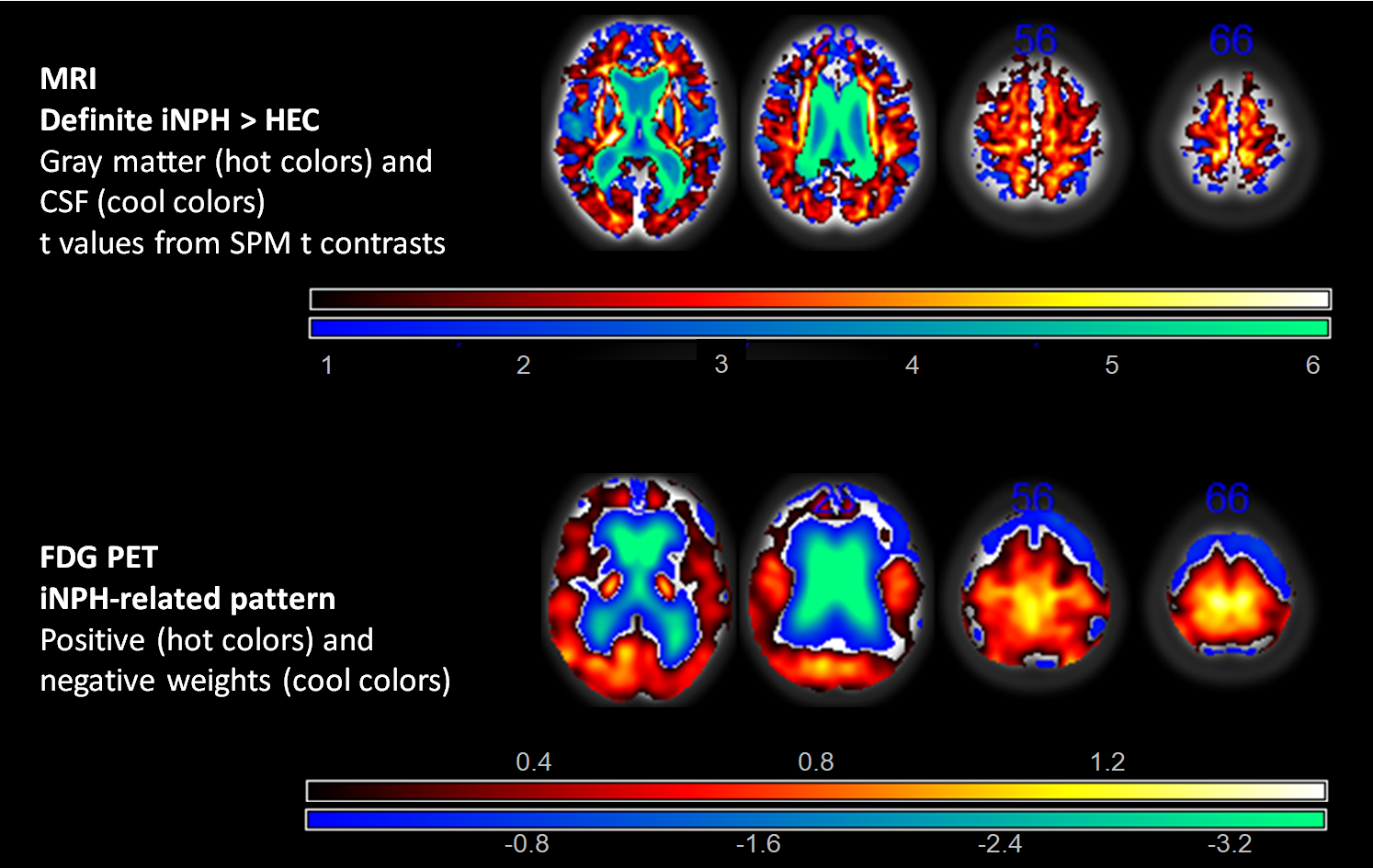
**Comparison between the relative increases of CSF and gray matter in definite iNPH (obtained from MRI) and the FDG PET-derived iNPHRP in the same subjects.** Top, t values from group comparisons of CSF and gray matter volume between definite iNPH patients and HEC (separate SPM12 t tests). Bottom, iNPHRP positive and negative voxel weights.
